# Intraventricular Meningiomas: Clinical-Pathological and Genetic Features of a Monocentric Series

**DOI:** 10.3390/curroncol29010017

**Published:** 2022-01-02

**Authors:** Serena Ammendola, Michele Simbolo, Chiara Ciaparrone, Paola Chiara Rizzo, Maria Caffo, Giampietro Pinna, Francesco Sala, Aldo Scarpa, Valeria Barresi

**Affiliations:** 1 Dipartimento di Diagnostica e Sanità Pubblica, Università degli Studi di Verona, 371234 Verona, Italy; serena.ammendola@univr.it (S.A.); michele.simbolo@univr.it (M.S.); chiara.ciaparrone@univr.it (C.C.); paolachiara.rizzo@studenti.univr.it (P.C.R.); aldo.scarpa@univr.it (A.S.); 2 Department of Biomedical and Dental Sciences and Morphofunctional Imaging, Section of Neurosurgery, University of Messina, 98122 Messina, Italy; mcaffo@unime.it; 3 Unit of Neurosurgery, Department of Neurosciences, Hospital Trust of Verona, 37126 Verona, Italy; giampietro.pinna@aovr.veneto.it; 4 Department of Neurosciences, Biomedicines and Movement Sciences, Institute of Neurosurgery, University of Verona, 37126 Verona, Italy; francesco.sala@univr.it; 5 ARC-Net Research Centre, University and Hospital Trust of Verona, 37134 Verona, Italy

**Keywords:** meningioma, intraventricular, *NF2*, *SMARCB1*, tumor mutational burden, TMB

## Abstract

Intraventricular meningiomas (IVMs) are rare (0.5–5%) and usually low-grade (90% grade I) brain neoplasms. Their recurrence rate is lower than that of extra-axial meningiomas, but their surgical resection can be burdened with life-threatening complications, which represent the major cause of the reported 4% mortality. The aim of this study is to characterize the molecular portrait of IVMs to identify potential therapeutic targets. For this, we explored mutations and copy number variations (CNV) of 409 cancer-related genes and tumor mutational burden (TMB) of six cases, using next-generation sequencing. Five IVMs were grade I and one was grade II; none recurred, in spite of partial surgical resection in one case. *NF2* mutation was the only recurring alteration and was present in three of the six IVMs, in association with *SMARCB1* mutation in one case. None of the cases was hypermutated (TMB > 10 mutations/Mb). *NF2*-mutant progressing or recurring IVMs could potentially be treated with targeted therapies applied to other *NF2*-mutant tumors, as an alternative to surgery or radiosurgery, while in view of their low TMB they are unlikely candidates to immune check-point inhibition.

## 1. Introduction

Meningiomas mostly arise in the cerebral meninges (>80%) and are among the most frequent tumors of the central nervous system, accounting for approximately 38% of brain neoplasms overall [1]. They are classified into three histological grades of malignancy, with grade I being about 80% and grades II and III representing 20–25% and 1–6%, respectively [1]. The histological grade, together with the extent of surgical resection, represents a major prognostic factor of recurrence risk in patients with these tumors [1]. 

Irrespective of the histological grade, most meningiomas (55%) exhibit *NF2* alterations, while *NF2* wild-type meningiomas may harbor mutations in *TRAF7*, *KLF4*, *PIK3CA* or *SMO* [2]. *NF2*-altered tumors are mainly localized at the convexity or in the lateral and posterior skull base; *SMO*-mutated meningiomas arise in the olfactory groove, while those with *AKT1* and *PIK3CA* mutations are mostly found in the anterior and middle skull base [3]. With increasing histological grade, meningiomas accumulate copy number alterations, among which *CDKN2A* homozygous deletion or 1p, 6q, 14q, 18q and 22q loss of heterozygosity (LOH) are prognostically significant [4].

The recommended treatment for symptomatic patients with meningioma is surgery, followed by fractioned radiotherapy in grade II/III or in partially resected grade I tumors [5]. Radiosurgery is indicated as an alternative to surgery or radiotherapy [5]. Although the role of pharmacotherapy is currently ill-defined [5], a phase II trial is currently investigating the efficacy of targeted therapies in meningiomas harboring specific, targetable alterations, including *SMO/AKT/NF2/CDK* mutations (NCT02523014). In addition, the value of immune check point inhibition is being explored in recurrent, residual or metastatic meningiomas (NCT03279692, NCT02648997).

Intra-ventricular meningiomas (IVMs) are a rare group of meningiomas (0.5–5%) originating in the cerebral ventricles [6,7,8,9]. According to a recent review of 625 cases, they mostly localize in the lateral ventricles, are mainly classified grade I (89.8%) and have a lower recurrence rate than the extra-axial meningiomas (2% vs. 7–25% for grade I; 14% vs. 29–52% for grade II; and 31% vs. 50–94% for grade III) [1,10]. The reported mortality of 4% is mostly (65%) associated with post-surgical complications (hematoma in surgical site, tumor bleeding, surgical site infection, pulmonary embolism, bronchopneumonia and abscess) and less frequently with tumor progression/recurrence [10]. The limited knowledge of their molecular profile comes from a unique previous study, which analyzed 17 IVMs using next-generation sequencing of a panel of 130 cancer related genes frequently mutated in brain tumors [8]. *NF2* mutation was present in 47% of cases and represented the only recurring genetic alteration in these tumors, while *SMARCB1*/*A4* mutations were found in only one recurring grade II IVM [8]. Five IVMs were included in another study performing whole-genome sequencing of 775 meningiomas; two cases had *NF2* mutation, one featured *SMO* mutation and no other mutations were found [11]. 

In the aim to clarify the molecular landscape of IVMs, in this study we analyzed the mutation and copy number variations (CNVs) of 409 genes and tumor mutational burden (TMB) in six cases. Knowledge of the genetic alterations may be useful to identify potential targeted therapies, which could be useful to reduce the life-threatening complications of surgery.

## 2. Materials and Methods

### 2.1. Cases

Among 1193 meningiomas operated in our institution between January 2011 and July 2021, six (0.5%) were IVMs and were considered in this study.

Information on the localization, extent of surgical resection, post-surgical complications, adjuvant treatments and development of recurrences was retrieved using operatory registries and clinical records. The extent of surgical resection was classified as total or partial. Recurrence was defined as the identification of a tumor in the site of previous surgery by means of computerized tomography or magnetic resonance imaging. RFS was the length of survival to the detection of a recurrent tumor or to the last follow-up time.

All cases were classified according to WHO 2016 criteria [1], after the revision of histological slides. 

### 2.2. Ethical Issues

This study was approved by Comitato Etico per la Sperimentazione Clinica delle province di Verona e Rovigo (protocol n. 40400, 19 July 2019).

### 2.3. Mutational and Copy Number Variation Status of 409 Cancer Genes

DNA was obtained from 10 formalynformalin-fixed, paraffin-embedded (FFPE) consecutive 4-μm sections using the QIAamp DNA FFPE Tissue Kit (Qiagen, Hilden, Germany) and qualified as previously reported [12]. The targeted next-generation sequencing (NGS) panel used was the Oncomine Tumor Mutational Load (TML) assay (ThermoFisher, Waltham, MA, USA), which covers 1.65 Mb of genomic space for the assessment of tumor mutational burden and explores all exons of 409 cancer-related genes for mutational and copy number assessment.

Sequencing was performed on Ion Torrent platform using 20 ng of DNA for each multiplex PCR amplification and subsequent library construction. The quality of libraries was evaluated using the Agilent 2100 Bioanalyzer on-chip electrophoresis (Agilent Technologies. Santa Clara, CA, USA). Libraries were clonally amplified by emulsion PCR with Ion OneTouch OT2 System (Thermofisher, Waltham, MA, USA), and sequencing was run on Ion Proton (Thermofisher, Waltham, MA, USA) loaded with Ion PI Chip v3.

Torrent Suite Software v.5.10 (Thermofisher, Waltham, MA, USA) was used for data analysis, including alignment to the hg19 human reference genome and variant calling. Filtered variants were annotated using a custom pipeline based on vcflib [13] (https://github.com/ekg/vcflib, accessed on 16 November 2021), SnpSift [14], variant effect predictor (VEP) [15] and NCBI RefSeq database. Additionally, alignments were visually verified with the integrative genomics viewer (IGV) v2.3 [16] to confirm the presence of identified mutations.

CNVs were assessed using OncoCNV v6.8 [17], comparing the BAM files obtained from tumor samples with those obtained from blood samples of four healthy male subjects.

The software includes a multi-factor normalization and annotation technique enabling the detection of large copy number changes from amplicon sequencing data and permits to visualize the output per chromosome.

TMB and mutational spectrum were assessed using the Oncomine TML 5.10 plugin available on IonReporter software (Thermofisher, Waltham, MA, USA). Default Modified parameters were used to exclude se-quencing artefacts. Specifically, a threshold of at least 20 reads and an allelic frequency of 10% of variant were used for mutation calling. TMB is expressed as the number of mutations per Mb (muts/Mb), where mutations include non-synonymous missense and nonsense single nucleotide variants (SNVs), plus insertion and deletion variants (InDels) detected per Mb of exonic sequences.

## 3. Results

### 3.1. Cases

**Figure 1:**
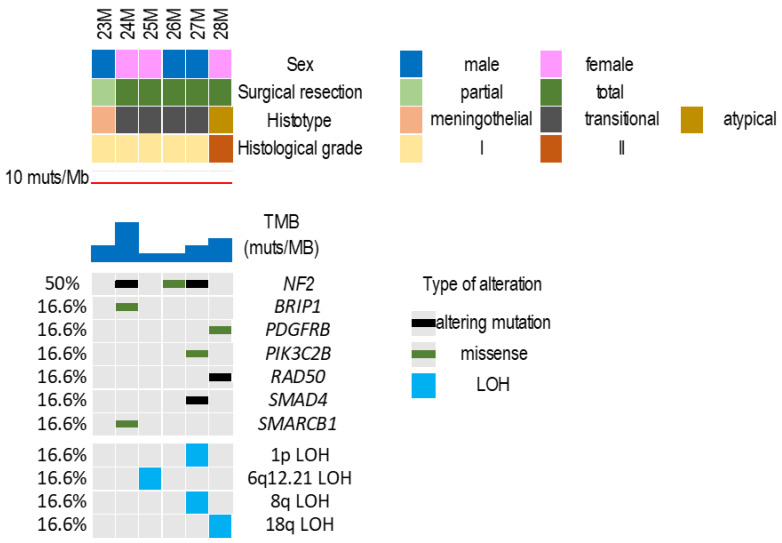
Clinical-pathological features and genomic landscape of six IVMs. The matrix shows seven genes that were altered at sequencing analysis, and the main chromosomal alterations.

The clinical and pathological features of the six IVMs are summarized in Figure 1 and detailed in Table 1.

**Figure 2:**
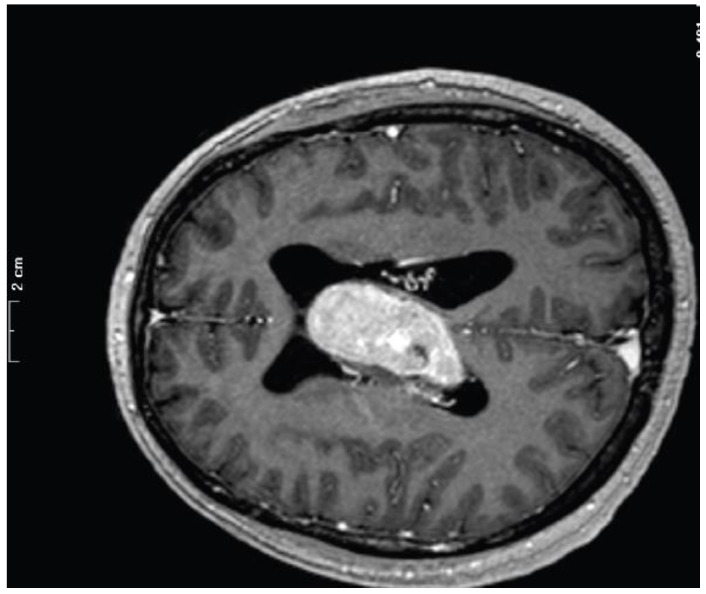
Magnetic resonance imaging of case 23M, showing IVM within the third and lateral ventricles.

Three cases were from male and three were from female patients (age range: 25–70 years; mean age: 47 ± 18 years; median: 46 years). Three meningiomas were localized in the left lateral ventricle, two were in the right lateral ventricle, and one was involved the third and the lateral ventricles (Figure 2). Four patients had surgery-related complications.

With the exception of this latter case, which had partial resection followed by gamma-knife radiodurgery, all IVMs had gross total resection and did not receive any adjuvant treatments. Five patients were followed-up and none developed recurrences during a time ranging between 7 and 72 months.

The histotype was transitional in four cases, meningothelial in one and atypical in another (case 28M). This latter case was classified atypical based on the presence of macronucleoli, spontaneous necrosis and small cells with high nuclear/cytoplasmic ratio.

### 3.2. Mutational and Copy Number Variation Status of 409 Cancer Genes

Four of the six IVMs had at least one gene mutation; two cases (23M and 25M) had no mutations in any of the 409 genes analyzed (Figure 1; Table S1).

The CNV status was estimated for all 409 genes using sequencing data.

Three meningiomas (50%) had *NF2* alterations, consisting in truncating (24M and 27M) or frameshift (26M) mutations. In one case (24M), *NF2* alteration co-occurred with *SMARCB1* and *BRIP1* mutations, and in another (27M), it associated with *PIK3C2B* and *SMAD4* mutations. One *NF2* wild type meningioma (28M) had *PDGFRB* and *RAD50* mutations.

We did not identify recurrent CNVs. One transitional meningioma (27M, 17%) had 1p and 8q LOH, and the atypical meningioma had 18q LOH. One case (25M) had 6q12.21 LOH.

### 3.3. Tumor Mutational Burden

The number of mutations/Mb in the 6 IVMs ranged between 0.87 and 5.13 (median: 1.79). Using a cut-off value of 10 mutations/Mb, none of the cases were hypermutated. Applying Mann–Whitney test, the TMB counts in this cohort were significantly lower than those found in 22 atypical extra-axial meningiomas in our previous study [1] (*p* = 0.0004). Even the grade II IVM (case 28M) had a lower TMB than all 22 cases previously assessed.

The highest TMB (TMB: 5.13 muts/Mb) was found in the *NF2*/*SMARCB1*-mutated meningioma (24M).

## 4. Discussion

The knowledge of the molecular landscape of meningiomas has progressively increased over the last years, providing novel prognostic markers and potential therapeutic targets for these tumors [4,18,19]. Several studies have clarified that meningioma is not a unique entity but rather a group of tumors showing diverse genetic alterations according to their histotype and anatomical location [2,3]. Indeed, skull-based and non-skull-based meningiomas exhibit different molecular features, and even among skull-based meningiomas, variation exists in relation to medial or lateral, anterior or posterior localization [3].

IVMs are a rare group of meningiomas defined by their localization within the intra-cerebral ventricles. In agreement with previous literature [10], in the present series we did not observe a female predilection and found a preferential location in the lateral ventricles, with slight predominance of left-sided tumors. Consistent with previous observation that IVMs are indolent tumors [10], all but one were grade I and none recurred. Although one meningioma had only partial resection, no recurrence was observed likely as an effect of the following radiosurgery treatment. Four cases in this series had transitional histotype as the majority of the IVMs previously characterized by genetic analyses [8,11]. The only grade II meningioma was classified atypical due to minor criteria and lacked brain invasion or high mitotic count. As expected, and in relationship with the peculiar site of these tumors, most patients had surgery-related complications.

In accordance with other studies, which reported that about half IVMs are *NF2*-mutant and that this is the only recurrent mutation in these tumors [8,11], *NF2* mutation represented the most frequent genetic alteration in this cohort and was found in 3/6 (50%) IVMs. All three *NF2*-mutated meningiomas had transitional histotype, confirming the previously reported association between *NF2* mutations and fibrous, transitional or atypical histotypes [20].

One *NF2*-mutant IVM had co-occurring mutations in *PIK3CD2B* and *SMAD4*. *PIK3CD2B* encodes for PI3KC2β protein that is involved in *PI3K-AKT-mTOR* pathway. Mutations in this gene were reported in several other tumor types, but their significance in tumorigenesis is still unclear [21]. *SMAD4* is a tumor-suppressor gene, acting as a downstream regulator of the *TGF-β* pathway [22]. Its mutations were reported in colorectal carcinomas with worse prognosis [22] but were undescribed in meningiomas. The *NF2/PIK3CD2B/ SMAD4* mutant IVM featured 1p LOH, which was previously linked to meningioma recurrence [23].

Another *NF2*-mutated meningioma featured *SMARCB1* R386H and *BRIP2* mutations. *SMARCB1* R386H mutation was reported in 2.4% of meningiomas, mostly in association with *NF2* mutations (73.6%) [11]. *SMARCB1*-*NF2* mutant meningiomas account for 6.8% of all *NF2*-mutant meningiomas and are preferential localized in the midline dura (falx) or in the anterior convexity [11]. The present case demonstrates that *NF2/SMARCB1*-mutant meningiomas are not exclusive to midline sagittal localization but may be also intra-ventricular. In the previous molecular study on IVMs, *SMARCB1* R377H mutation was considered prognostically unfavorable as it was found in an atypical, relapsing, meningioma [8]. The mutated case in our series, having an uneventful 48 months follow-up, seems to disprove that *SMARCB1* mutations are associated with an increased risk of relapse of IVMs. We did not find any other genetic alterations in the third *NF2*-mutant meningioma in this cohort.

Concerning the three *NF2*-wild type IVMs, none had mutations in *AKT1*, *PIK3CA* or *SMO* genes, which are typically altered in skull base meningiomas, [19,24,25]. One case, histologically classified atypical (grade II), had *PDGFRB* and *RAD50* mutations. RAD50 is part of the MRE11-RAD50-NBS1 complex, which plays a crucial role in sensing and repair of DNA damage [26]. Since in vitro studies demonstrated that mutant RAD50 may sensitize tumor cells to radiation, we may speculate that *RAD50* mutant meningiomas may be more sensitive to radiotherapy [26]. 18q LOH found in this case was previously associated with grade II meningiomas and with increased recurrence risk [4]: nonetheless, the short follow-up time in this patient did not allow drawing any significant conclusions on its prognostic significance in IVMs.

The two other *NF2*-wild type meningiomas had no mutations in any of the 409 genes analyzed. However, one of these two cases had a heterozygous deletion at chromosome 6q12.21, where *MAP3K7* gene is located. This microdeletion of 6q was previously reported in prostatic carcinoma in association with a worse prognosis [27,28]. The short follow-up time in this patient did not allow establish whether 6q12.21 microdeletion is prognostically informative in IVMs.

Among the ongoing clinical trials in patients with meningiomas, two are assessing the efficacy of mTOR or FAK inhibitors in recurring or progressing cases (NCT02523014, NCT03071874). The presence of *NF2*-mutations in IVMs of this and other series [8,11] suggests that they may be potentially targeted with these drugs. Other trials are testing the efficacy of immune check point inhibitors in recurrent or residual or metastatic meningiomas (NCT03279692, NCT02648997), and there is some evidence that hypermutated meningiomas (i.e., having > 10 mutations/Mb) may be responsive to these therapies [29]. Herein, we first assessed TMB in IVMs and found no hypermutated cases. In addition, the median TMB in this cohort was significantly lower than that previously found in cohorts of high grade meningiomas (1.79 vs. 5.32 and 9 mutations/Mb in other studies) [4,30], suggesting a correlation between TMB and the histological grade and biological aggressiveness of these tumors. The only grade II meningioma in this cohort had a TMB of 2.28 mutations/Mb, which confirms that meningiomas classified atypical on minor criteria only have low mutation counts [4].

## 5. Conclusions

In conclusion, this study expands the knowledge on the molecular features of IVMs, confirming the lack of recurrent alterations different from *NF2* mutations. Noteworthy, none of the cases had gene mutations typically observed in skull base meningiomas and involving *AKT1*, *PIK3CA* and *SMO*, while one case had co-occurring *NF2* and *SMARCB1* mutations previously found in sagittal meningiomas. Differently from that previously suggested in another series of IVMs, *SMARCB1* mutation was not associated with a higher aggressiveness in this cohort.

This is the first study to assess TMB in IVMs, and the low mutation counts that we found suggest that check point inhibition is likely ineffective in these tumors. However, inoperable patients or those with residual or progressing disease could be potentially benefit of molecular therapies targeting *NF2* mutations.

## Figures and Tables

**Table 1:** Clinical-pathological features and TMB of six IVMs.

Case	Age	Sex	Site	Surgery	Histotype	Post-Surgical Complications	Adjuvant Treatment	Recurrence (RFS)	TMB (muts/Mb)
23M	45	M	III, LV, RV	Partial	meningothelial	respiratory failure, hydrocephalus, and surgical site infection	GK	no (40)	1.87
24M	47	F	LV	Total	transitional	none	None	no (48)	5.13
25M	25	F	RV	Total	transitional	pneumatocephalus	None	no (12)	0.87
26M	70	M	RV	Total	transitional	pneumatocephalus	None	no (72)	0.86
27M	66	M	LV	Total	transitional	hematoma in surgical site	None	not available	1.71
28M	31	F	LV	Total	atypical	none	None	no (7)	2.28

M: male. F: female. RFS: recurrence-free survival. III: third. LV: left ventricle. RV: right ventricle. GK: gamma-knife.

## Data Availability

Data will be available upon request.

## Supplementary Materials

The following are available online at https://www.mdpi.com/article/10.3390/curroncol29010017/s1, Table S1: List of somatic and germline mutations identified in all samples.
